# Spouse’s coping strategies mediate the relationship between women’s coping strategies and their psychological health among infertile couples

**DOI:** 10.1038/s41598-023-37380-x

**Published:** 2023-07-01

**Authors:** Marzie Reisi, Ashraf Kazemi, Mohammad Reza Abedi, Naser Nazarian

**Affiliations:** 1 School of Nursing and Midwifery, Isfahan University of Medical Sciences, Isfahan, Iran; 2 Nursing and Midwifery Care Research Center, School of Nursing and Midwifery, Isfahan University of Medical Sciences, Hezarjerib AV., Isfahan, Iran; 3 Counseling Department, School of Psychology and Education Sciences, University of Isfahan, Isfahan, Iran; 4 Social Sciences Department, University of Isfahan, Isfahan, Iran

**Keywords:** Patient education, Psychology, Health care

## Abstract

Social problems and suffering from the treatment process for infertile couples, especially for women, require the couples to cope with them to balance the infertility crisis. According to the close interactions of the couples with each other, the objective of the present study was to explore a theoretical framework for the relationships between women’s coping strategies, spouses’ coping strategies, and women’s psychological health in infertile couples who were candidates for assisted reproductive technology (ART). This cross-sectional study was carried out on 212 couples undergoing ART. The couples’ coping strategies were evaluated using a validated self-report questionnaire. The women’s psychological health was assessed using a 21-item stress, anxiety, and depression scale (DASS-21). Statistical analysis was performed using the plug-in application PROCESS macro for SPSS. The direct effect of the women’s self-blame and self-focused rumination strategies (*p* < .0001), and the indirect effect of the women’s self-blame on stress and depression by mediating spouses’ self-blame and self-focused rumination strategies, was significant. The indirect effect of the women’s self-focused rumination on the anxiety and depression levels by mediating spouses’ self-blame strategy was significant. The women’s self-blame and self-focused rumination strategies had a negative effect on the women’s psychological health who were undergoing ART. This negative effect was mediated by the spouse’s coping strategies.

## Introduction

Infertility, with a prevalence of approximately 15%^1^, encounters couples with numerous social problems, such as the increased probability of domestic violence^2,3^, divorce^4^, polygyny in some countries^5^, social isolations^6,7^, and reduced life quality of infertile couples^8^. Infertility may affect a couple's mental health^9,10^; however, assisted reproductive technology (ART) procedures, such as ovarian induction, receiving eggs, and embryo transfer, are mainly carried out on women and expose them to the resulting complications^11^. Therefore, injuries from these treatments and the adverse psychological effects of infertility and its treatment are more prevalent in women than in men^12^.

Moreover, infertility is often attributed to women^13^: therefore, women are under social pressure for infertility more than men^3,14^. Although using an ART has apparent successes, suffering from infertility stigma^4,13^, stress from ART processes^8,9^, and the probability of treatment failure have turned infertility into a crisis. Preventing the negative psychological effects of infertility on women requires balancing the crisis by using effective and efficient coping strategies among infertile couples^15^.

Coping strategies are ways an individual employs to manage life crises and include focusing on the problem or the emotions^16^; however, maladaptive coping strategies may not positively affect psychological health in couples who undergo ART^17,18^.

The association between the anxiety level in women undergoing infertility treatment and maladaptive coping strategies has been previously reported. Peloquin et al. reported that the self-blame strategy predicted anxiety and depression symptoms in both men and women^19^. However, one study showed that self-blame in women was related to higher marital quality and psychological health^20,21^.

Infertility experiences accompanying stigma and social suffering^4,13^, that provide the field for selecting the coping strategy may explain these differences. It is believed that the severity of the socio-psychological burden of infertility depends on the importance of fertility in the social context^8,22^.

Zurlo et al. believe that the adequacy of coping strategies in modifying the crisis is situation-dependent. They reported that, against all odds, the problem-oriented coping strategy intensified the effect of problem-solving on the adverse effects of social anxiety^18^. These contradictory results might originate from ignoring interdependence in the dyadic relationships of infertile couples since the way each couple manages infertility affects their marital relationship^23^.

The shared nature of the infertility experience raises the probability of a correlation between the coping strategies of each infertile couple and its impact on their mental health. Identifying the interactive effect of coping strategies in couples is essential for developing counseling programs for women undergoing infertility treatment.

Therefore, the aim of this study was to explore a theoretical framework for the relationships between women’s coping strategies, spouses’ coping strategies, and women’s psychological health in infertile couples who were candidates for ART. The study hypotheses were: (1) women’s coping strategies may influence their spouse’s coping strategies, (2) women’s coping strategies may influence their psychological health, and (3) spouses’ coping strategies may mediate the relationship between women’s coping strategies and psychological health.

## Materials and methods

This cross-sectional study was conducted on 212 couples under ART in the Iran-Isfahan infertility center from January 2019 to May 2019. The study was reviewed and approved by the Ethics Committee of Isfahan University of Medical Sciences (IR.MUI.NUREMA.REC.1400.019). The sample size was calculated considering the confidence coefficient of 95% (1.96) and test power of 80% (0.84). During calculation, the correlation coefficient between the coping strategy score and mental health variables was considered 0.2. The number of samples was calculated as 190 couples, which increased to 212, considering the potential 10% sample attrition. The following formula was used to estimate the number of samples: n = [(z_1_ + z_2_)^2^ (1 − r^2^)/r^2^] + 2.

The inclusion criteria included using one’s own oocyte for ART and stressful crises based on Holmes- Rahe-Stress-Scale. Convenience sampling was performed for the ART candidate couples before starting the ovarian stimulation protocol.

A preliminary interview was implemented, and invited couples were ensured that their participation or non-participation would not affect their treatment process and that their information would be completely confidential. They were then provided with the necessary explanations about the study objectives, and informed consent was obtained from the eligible couples.

Inclusion criteria included using the information recorded in the couple’s file and completing the Holmes Rahe scale as a self-report. Only couples who both agreed to enter the study and complete the questionnaires were included in the study.

A total of 230 couples were invited to participate in the study by one of the researchers while visiting the infertility center and receiving ovulation stimulation drugs; 212 couples accepted to participate.

### Instruments

The couple coping strategies and women’s psychological health were assessed using self-report questionnaires. The coping strategies were evaluated using a 20-item scale developed using two questionnaires^24,25^. This questionnaire was designed on a Likert scale from rarely (1) to frequently (4), with five domains including self-blame (4 items), self-focused rumination (4 items), goal replacement (4 items), avoidance (4 items), and active confronting (4 items) strategies.

The reliability of the instrument was measured using a pilot study on 15 eligible couples via a retest method with 3-week intervals. The intra-class correlation was calculated to determine the stability of the questionnaire.

Internal reliability of the questionnaire with Cronbach’s alpha of 0.86 and repetition with an Intra-Cluster correlation index of 0.72 was confirmed. As the psychological health variables, the levels of depression, anxiety, and stress in women were evaluated using a 21-items depression, anxiety, and stress scale (DASS-21) with Cronbach’s alpha of 0.0.77 for depression, 0.79 for anxiety, and 0.78 for stress^26^.

### Statistical analysis

Data analysis was performed using SPSS software (version 19) and plug-in application PROCESS macro v 3.4 by Hayes. The linear regression was used to investigate the relationship between women’s and their spouses’ coping strategies and women’s depression, anxiety, and stress levels.

To determine the potential confounding variables, Pearson and Spearman correlation coefficients were used, and variables correlated with women’s depression, stress, anxiety, and coping strategies entered the regression model as covariant. The statistical significance of the mediating variable (spouses’ coping strategies) was examined over 10,000 bootstrap samples. This method generated an estimate of the indirect effect, including 95% confidence intervals. When zero was not within the 95% confidence limits, it was concluded that the indirect effect was significantly different from zero.

### Ethics approval and consent to participate

All procedures performed on participants were in accordance with the ethical standards of the Isfahan University of Medical Sciences, and informed consent was obtained from all participants.

## Results

Data analysis was performed on 212 couples with 100% participation. The results showed that participants’ mean (standard deviation) of infertility duration was 6.0 (4.3) years. In 107 (50.47%) couples, the main cause of infertility was the female factor; in 80 (37.74%), it was the male factor. Twenty-five (11.79%) couples had unexplained infertility. The couples’ profiles and the level of their coping strategies are shown in Table 1.

**Table 1:** Demographic characterizes and main variables.

	Mean (SD) or number (%)	
	Women	Spouses
Number	212	212
Age	32.2 (4.8)	36.6 (5.0)
Educational level (%)		
Less than high school	23 (11.3)	30 (14.1)
High school diploma	157 (74.1)	161 (76.0)
University degree	31 (14.6)	21 (9.9)
Coping strategies		
Self-blame	8.3 (3.4)	7.1 (3.2)
Self-focused rumination	10.4 (3.9)	8.5 (3.5)
Goal replacement	10.2 (3.5)	10.8 (3.3)
Avoidance	10.1 (3.1)	10.3 (3.0)
Active confronting	8.4 (3.4)	7.0 (3.1)
Psychological health		
Depression	10.7 (5.7)	–
Anxiety	9.9 (3.3)	–
Stress	14.0 (5.6)	–

*SD* standard deviation.

Assessments of correlation coefficients (Pearson and Spearman correlation coefficient) showed that the women’s coping strategies were associated with their age, education level, infertility factor, and infertility duration (the results were not presented); therefore, these variables entered the regression model as covariant variables.

Independent of potential confounders, the spouses’ self-blame and self-focused rumination strategies were related to the women’s self-blame and self-focused rumination strategies. Moreover, avoidance strategy in men was related to the women’s self-focused rumination strategies (Table 2).

**Table 2:** The relation between partners’ coping strategies (212 couples).

	Spouse’s coping strategy (CS)														
	Self-blame			Self-focused rumination			Goal replacement			Avoidance			Active confronting		
	Beta	CI 95%		Beta	CI 95%		Beta	CI 95%		Beta	CI 95%		Beta	CI 95%	
Age	.01	− .01	.02	.01	− .01	.02	.13	− .02	.19	− .02	− .01	.005	− .11	− .01	.03
Education level	.04	− 03	.04	.02	− .04	.04	− .04	− .07	.15	.15	− .01	.05	− .09	− .06	.02
Duration of Infertility	− .06	− .03	.02	− .01	− .02	.04	− .10	− .18	.31	.04	− .02	.01	− .05	− .01	.08
Male factor infertility	.02	− .05	.08	.03	− .05	.10	.24*	.09	.34	.06	− .04	.07	.09	− .11	.34
Female factor infertility	.03	− .02	.06	.01	− .02	.04	.11	− .04	.22	.21*	.11	.34	.03	− .01	.05
Unexplained infertility	.01	− .06	.05	.07	− .03	.11	.09	− .11	.19	.05	− .03	.06	.01	− .02	.03
Women’s CS															
Self− blame	.25*	.13	..29	.30**	.17	.41	− .14	− .16	.11	− .11	− .10	.02	− .03	− .27	.19
Self− focused rumination	.32**	.12	.53	.25*	.12	.44	.12	− .05	.32	.37**	.13	.47	− .02	− .27	.23
Goal replacement	.07	− .02	.11	− 17	.11	− .02	.11	− .02	.23	.02	− .14	.17	− .05	− .28	.14
Avoidance	.11	− .02	.15	.09	− .05	.23	.09	− .01	.32	.05	− .14	.23	− .11	− .12	.09
Active confronting	.12	− .09	.18	.03	− .21	.15	− .09	− .12	.11	39	− .12	.17	.11	− .02	.23

*CS* copping strategies. *p < 0.05; **p < .01; ***p < .001.

Significant values are in bold.

The anxiety and stress levels were related to the women’s active confronting strategy. The relationships between age and educational level and depression, anxiety, and stress levels were significant. Independent of potential confounders, the depression, anxiety, and stress levels were positively related to women’s self-blame and self-focused rumination strategies and negatively related to women’s goal replacement strategy (Table 3). Evaluation of the relationship between spouses' coping strategies and women's psychological health showed that independent of potential variables, depression, anxiety, and stress levels were positively related to the spouses’ self-blame and self-focused rumination strategies (Table 4).

**Table 3:** The relation between women’s coping strategies and women’s psychological symptoms (212 women).

	Depression				Anxiety				Stress			
	R^2^Adj = .29 *p* < .0001, F = 10.79				R^2^_Adj_ = .24 *p* < .0001, F = 5.28				R^2^Adj = .30 *p* < .0001, F = 8.48			
	Beta	Sig	CI 95%		Beta	Sig	CI 95%		Beta	Sig	CI 95%	
Age	− .6	ns	− .41	.19	− .13	ns	− .53	.04	− .06	ns	− .42	.15
Education level	− .13	ns	− 2.32	.11	− .09	ns	− 1.92	.40	− .08	ns	− 1.82	.48
Duration of Infertility	− .10	ns	− 3.23	.49	− .03	ns	− 2.18	1.37	− .09	ns	− 3.04	.49
Male infertility	− .12	ns	− 6.54	.41	− .6	ns	− 4.75	1.90	− .15	**.03**	− 7.10	− .48
Female infertility	− .12	ns	− 6.45	.25	− .03	ns	− 3.66	2.27	− .08	ns	− 4.84	1.06
Unexplained infertility	− .01	ns	− 4.11	3.61	− .01	ns	− 3.99	3.17	.01	ns	− 3.65	3.69
Coping strategy												
Self-blame	.32	** < .0001**	.59	1.40	.27	** < .0001**	.37	1.14	.25	** < .0001**	.39	1.22
Self-focused rumination	.17	**.007**	.13	.83	.19	**.004**	.16	1.52	.50	**.006**	.14	.85
Goal replacement	− .20	**.004**	− 1.02	− .19	− .16	**.04**	− .05	− .11	− .18	**.01**	− .95	− .12
Avoidance	.04	ns	− .34	.61	.07	ns	− .21	.67	.01	ns	− .47	.48
Active confronting	.13	ns	− .40	.80	.15	**.04**	.03	.81	1.25	**.003**	.22	1.06

*CSs* coping strategies, *ns* non-significant.

Significant values are in bold.

**Table 4:** The relation between spouse’s coping strategies (CSs) and women’s psychological symptoms (212 couples).

	Depression				Anxiety				Stress			
	R^2^_Adj_ = .19 *p* < .0001, F = 4.29				R^2^_Adj_ = .19 *p* < .0001, F = 3.96				R^2^_Adj_ = .17 *p* < .0001, F = 4.25			
	Beta	sig	CI 95%		Beta	sig	CI 95%		Beta	sig	CI 95%	
Age	− .18	**.01**	− .70	− .08	− .24	**.001**	− .76	− .19	− .19	**.008**	− .75	− .12
Education level	− .17	**.02**	− 2.83	− .24	− .17	**.03**	− 2.48	− .13	− .18	**.02**	− 3.55	− .33
Duration of Infertility	− .03	ns	− .29	.44	.06	ns	− .20	.47	− .02	ns	− .32	.41
Male infertility	− .13	ns	− 6.54	.41	− .12	ns	− 6.06	.77	− .20	**.01**	− 8.71	− 1.19
Female infertility	− .13	ns	− 6.06	.37	.02	ns	− 3.05	2.79	− .08	ns	− 5.34	1.63
Unexplained infertility	.04	ns	− 3.45	5.83	.04	ns	− 2.48	5.36	.06	ns	− 2.52	6.89
Spouse’s CSs												
Self-blame	.18	**.03**	.05	1.13	.18	**.03**	.05	1.02	.17	**.03**	.04	1.05
Self-focused rumination	.18	**.04**	.02	1.02	.22	**.02**	.09	1.01	.19	**.04**	.03	1.07
Goal replacement	.06	ns	− .27	.64	.09	ns	− .17	.66	.08	ns	− .22	.71
Avoidance	− .01	ns	− .34	.61	− .01	ns	− .39	.34	.02	ns	− .47	.58
Active confronting	− .03	ns	− .61	.44	− .07	ns	− .71	.25	− .04	ns	− .64	.44

*CS* coping strategies, *ns* non-significant.

Significant values are in bold.

**Figure 1:**
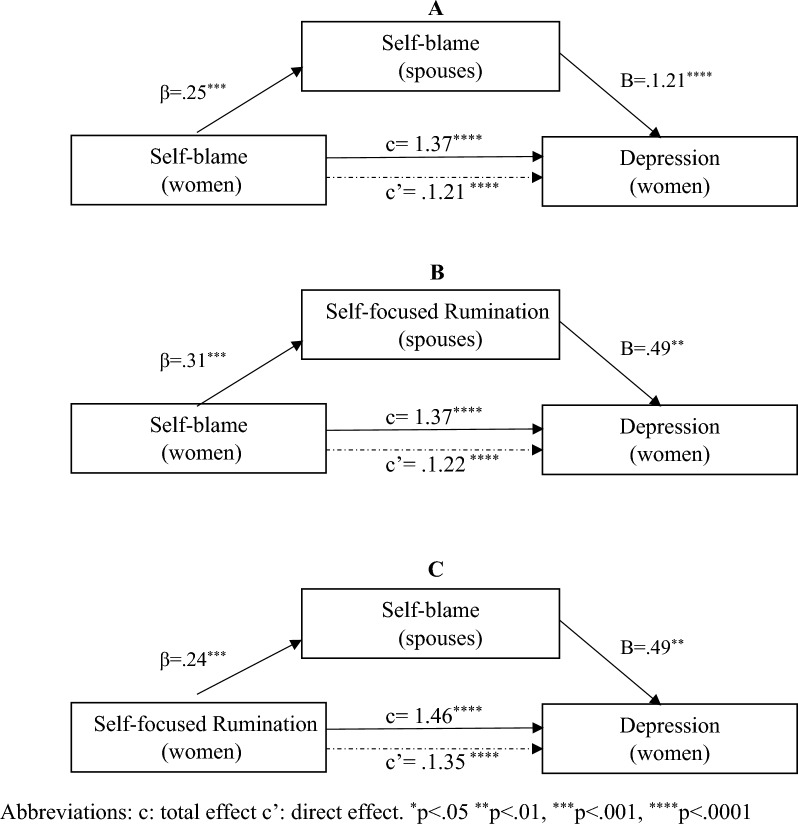
Mediation by spouse’s coping strategy of the association between women’s coping strategy and depression score.

**Figure 2:**
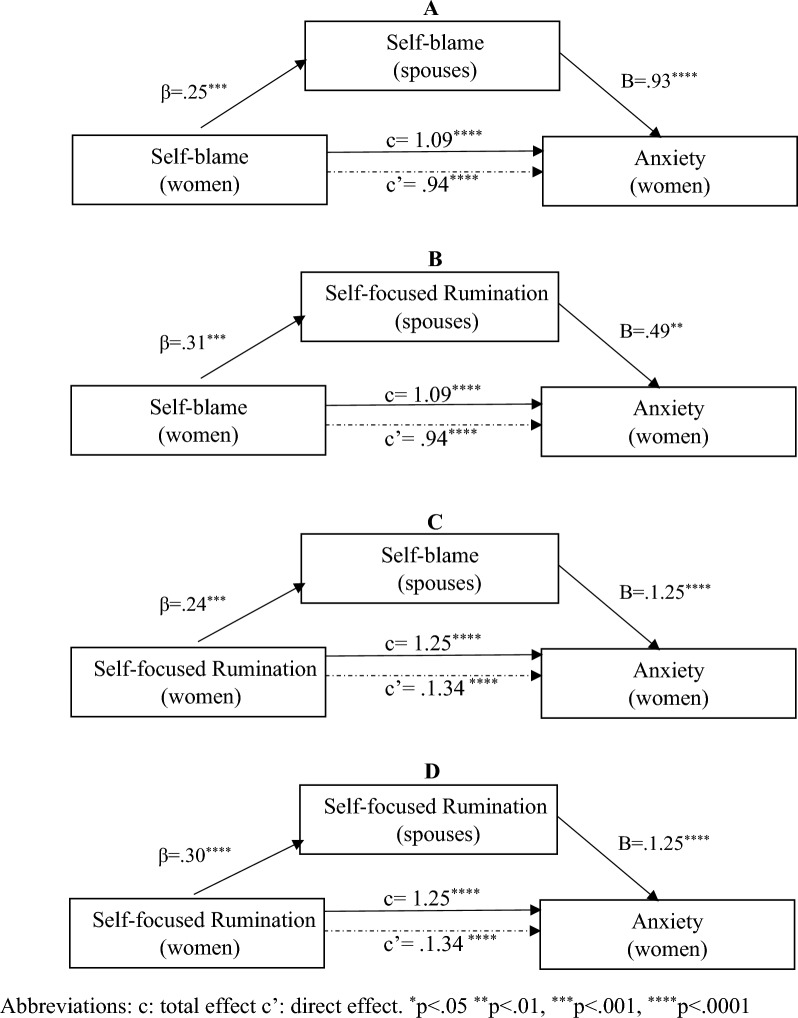
Mediation by spouse’s coping strategy of the association between women’s coping strategy and anxiety score.

**Figure 3:**
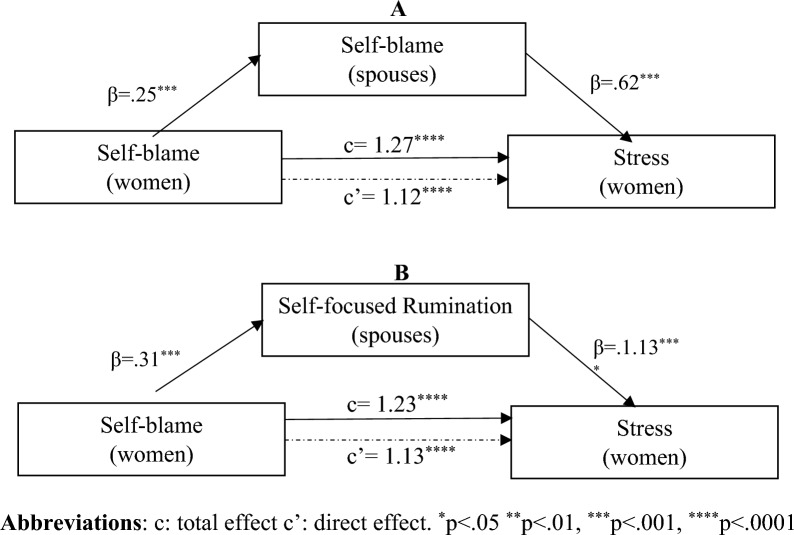
Mediation by spouse’s coping strategy of the association between women’s coping strategy and stress score.

The total and direct effects of the women’s self-blame and self-focused rumination strategies on the women’s depression (Fig. 1), anxiety (Fig. 2), and stress (Fig. 3) levels were significant. The indirect effects of the women's self-blame strategy on depression, anxiety, and stress levels were mediated by spouses’ self-blame and self-focused rumination strategies. The indirect effects of the women’s self-focused rumination strategy on depression and anxiety levels were mediated by spouses’ self-blame strategy. In addition, the effect of the women’s self-focused rumination strategy on their anxiety level was mediated by spouses’ self-focused rumination strategy. The interaction between couples’ self-focused rumination strategy was significant (Table 5).

**Table 5:** Indirect effects of women’s coping strategies on psychological symptoms by mediating spouse’s coping strategies and interaction between couple’s strategies (212 couples).

					Indirect effects					
					Product of confidents		Bootstrapping			
Women’s CSs		Spouses’ CSs		Symptom	Point Estimate	SE	95% CI		Interaction	
							Lower	Upper	F	sig
Self-blame	→	Self-blame	→	Depression	.157*	.077	.032	.333	.26	ns
Self-blame	→	Self-focused Rumination	→	Depression	.154*	.075	.032	.333	.34	ns
Self-focused Rumination	→	Self-blame	→	Depression	.120*	.006	.001	.023	2.68	ns
Self-focused Rumination	→	Self-focused Rumination	→	Depression	.113	.065	− .001	.025	6.59	**.01**
Self-blame	→	Self-blame	→	Anxiety	.151*	.077	.025	.325	.66	ns
Self-blame	→	Self-focused Rumination	→	Anxiety	.154*	.069	.038	.306	.86	ns
Self-focused Rumination	→	Self-blame	→	Anxiety	.114*	.060	.009	.247	6.60	**.01**
Self-focused Rumination	→	Self-focused Rumination	→	Anxiety	.112*	.058	.014	.240	7.65	**.006**
Self-blame	→	Self-blame	→	Stress	.155*	.080	.027	.335	.17	ns
Self-blame	→	Self-focused Rumination	→	Stress	.160*	.076	.032	.338	.35	ns
Self-focused Rumination	→	Self-blame	→	Stress	.089	.054	− .015	.218	1.09	ns
Self-focused Rumination	→	Self-focused Rumination	→	Stress	.085	.060	− .022	.215	4.58	**.03**

*CSs* coping strategies, *SE* standard error, *CI* confidence interval.

*Significant.

Significant values are in bold.

## Discussion

The present study aimed to evaluate coping strategies’ interactive effect on women’s mental health in couples undergoing ART. The results showed that couples interacted with each other using the self-focused rumination strategy, and the spouse’s use of self-focused rumination and self-blame strategies mediated the effect of self-blame and self-focused rumination strategies on women’s depression, anxiety, and stress levels.

The first finding of the study revealed that self-blame and self-focused rumination coping in men was dependent on self-blame and self-focused rumination coping in women. Moreover, men’s avoidance coping was positively related to women’s self-focused rumination coping. Ozkan et al. reported that men and women used similar coping strategies for infertility^27^. Regarding gender differences in dealing with infertility, Alosaimi et al. reported that in Saudi Arabia, women and men faced different stress types due to infertility and used dissimilar methods to cope with them^28^.

In evaluating the relationship between the coping strategies of each spouse on women’s mental health, this study’s results indicated that women’s depression, anxiety, and stress levels had a positive correlation with the use of self-blame and self-focused rumination strategies. In addition, frequent use of the active confronting strategy was associated with increased anxiety and stress levels. However, the goal replacement strategy used by the women had an inverse relationship with depression, anxiety, and stress levels.

Moreover, these strategies’ negative impact on women’s mental health was mediated by men’s use of self-blame and self-focused rumination strategies. The negative impact of infertile women’s maladaptive coping strategies on their depression and anxiety has already been reported^18,29^.

Mirzaasgari et al. reported the relationship between self-blame strategy and rumination with anxiety and depression in women undergoing assisted reproductive treatments. However, unlike the results of the present study, in their study, goal replacement and active confronting strategies had an inverse relationship with the level of stress, anxiety, and depression^30^.

Peloquin reported higher levels of anxiety and depression in women who used the self-blame strategy more than others. This study also reported that the self-blame strategy in men was associated with less satisfaction with married life^19^. Another study reported higher suicidal thoughts and attempts in women who used the self-blame strategy^31^. Similarly, Zurlo et al. reported that the self-focused rumination strategy in infertile couples was associated with emotional problems and signs of depression^32^.

These studies reveal the negative impact of using these strategies on women’s mental health. While confirming the results of mentioned studies, the present study showed that the effect of the spouse’s maladaptive coping strategies mediated the negative effects of using these strategies by women.

The positive relationship observed between women’s active confronting strategy and their anxiety and stress levels confirms the results of studies showing that in uncontrollable crises such as infertility, this strategy is ineffective in modifying the crisis^33^. Contrary to this finding, Khalid et al. reported that active coping was inversely related to distress in infertile women^34^.

The contradictions in the results of this study might be related to the infertility taboo in some societies. The expression of feelings and infertility issues, which requires the disclosure of infertility, may be associated with tolerating the infertility taboo in these communities. On the other hand, it is probable that individuals with higher stress and anxiety levels will broadly use this strategy compared to others, which could not be evaluated in the present study and requires prospective studies.

The observed inverse relationship between women’s use of goal replacement strategy and the level of their psychological symptoms confirms the results of a study reporting that women undergoing ART had modified the infertility crisis by setting new life goals^35^.

Another study finding showed that, unlike couples’ self-focused rumination strategy, women’s self-blame strategy did not interact with the couple’s coping strategies. This finding could be explained in the traditional context of the society under investigation. In traditional societies such as Iran, women are primarily regarded as responsible for infertility^4^, and the acceptance of this issue by the couple can cause the husband to disregard the self-blame strategy while the wife uses it. Other studies have likewise shown that in traditional societies, women use the self-blame strategy against infertility more than men^20,21^ and attribute the responsibility of infertility to them^36^. Another study has similarly shown a positive relationship between women’s use of this strategy and marital satisfaction^23^.

Another finding of the present study indicated that, although couples’ use of self-blame strategy did not interact with each other, the indirect effect of women’s self-blame and self-focused rumination strategies was mediated by self-blame strategy, similar to self-focused rumination. This finding reveals that in order to improve the mental health of women undergoing ART, it is essential to emphasize spouses’ avoidance of maladaptive strategies such as self-blame and self-focused rumination. In addition, this finding confirms the results of Casu et al.’s study, which showed that the effect of couples’ social support was mediated by their coping strategies^37^. Zurlo et al. likewise reported that couples’ dynamic interaction was a significant predictor of infertile women’s mental health^38^.

Renzi et al. similarly showed that men’s higher capability to identify and describe their emotions was associated with their wives’ higher quality of life^39^. It is believed that in dynamic interaction, it is indispensable for couples to make an effort to reduce their partner’s stress by using appropriate strategies^40^. In addition to confirming this recommendation, the results of the present study emphasize that it is required for infertile couples not to share maladaptive coping strategies.

This study showed that the mental health of women undergoing ART is correlated with the couples’ coping strategies. However, the limitations of the present study should be taken into account in the interpretation of the results. The first limitation of the present study was its cross-sectional nature, which reduces the strength of the causal relationship between couples’ coping strategies and women’s mental health. Moreover, men’s mental health status may influence this interactive cycle, and it is suggested to be considered in future studies.

This study was conducted on infertile couples undergoing ART, and its results cannot be generalized to those frustrated with their infertility treatment or who do not intend to continue treatment. Furthermore, the generalizability of the study results to couples in the early stages of infertility treatment who have not yet entered the ART process is under question. Furthermore, considering that the inclusion criteria was the participation of both individuals, the results cannot be generalized to couples who did not accompany each other to receive the protocol.

In conclusion, this study showed that women’s self-blame and self-focused rumination strategies directly affect their mental health. These strategies have indirect harmful effects by mediating spouses’ self-blame and self-focused rumination strategies. Besides, the negative effects of infertility on the mental health of women undergoing treatment are mediated by their goal replacement strategy. This study confirms the interactive effect of couples’ coping on women's health, and it is suggested that its results be used in developing counseling programs focused on couples. Based on the present study, it is recommended that couples’ avoidance of maladaptive strategies be accentuated in the mental health promotion programs for women undergoing ART.

Based on this study results, it is suggested that the coping strategies of couples undergoing infertility treatment be evaluated to notify men of the possible impact of their coping behaviors on their wives’ mental health. Moreover, men should be advised to avoid self-blame and rumination strategies in order to maintain their spouse’s mental health.

## Data Availability

Data and material are available on request from the corresponding author.
